# Thiersch’s Method of Skin-Grafting

**Published:** 1889-04

**Authors:** 


### THIERSCH'S METHOD OF SKIN GRAFTING.

E. Plessing, of Leipzig, has contributed an interesting paper on this subject to the *Archiv für klinische Chirurgie*, Bd. 37, Hft. 1. Thiersch, it will be remembered, says that the healing of a granulating surface depends on two factors: *First*, in the changing of the soft, succulent blood-carrying granulation into the bloodless, dry, cicatricial papillæ, a result that causes a diminution of the surface and the drawing together of the adjacent parts. *Secondly*, a covering over of the contracted papillæ with epidermic cells. These factors, the contraction of the wound and the growth of the pellicle, occur together, within certain limits; and when these limits are reached the granulating surface becomes stationary and inactive.

It is the upper stratum of granulation tissue that causes the shrinking after ordinary skin grafting, and also the insecurity of the result. Thiersch removes this upper layer, and Maas says that it is necessary both to freshen up at the edges of the ulcer, and especially to remove the upper layer thoroughly, and to expose completely the lower one with its horizontal capillaries; between this lower layer and the transplanted flap thorough adhesion will take place, never to be disturbed by cicatricial contraction.

The method followed in the Leipzig Clinic is thus described by Plessing: The part from which the skin is taken is completely disinfected; any disinfectant may be used, but during the course of the operation a 6 to 1000 sterilized salt solution is used. All the soft granulations are then scraped away from the granulating wounds with a sharp spoon, the bleeding surface irrigated with the salt solution, sponged, covered with protective and compressed for five or ten minutes until bleeding ceases. The best results are obtained when the granulations are about six weeks old, and after their growth has been limited by repeated cauterizing and compression.

The wound being thoroughly prepared, the skin-grafting is begun. The skin of the arm and thigh is most often used. The skin must be free from fat, and well stretched by the left hand, the right hand carrying a razor with a long, wide, concave blade, held flat, and drawn slowly with a sawing motion through the upper layers of the skin; all this time the blade must be kept moist with the salt solution. The grafts are transferred immediately from the knife to the prepared surface; the blade is laid on the wound, and the edge of the graft is drawn over on to the wound by means of a probe; as the blade is withdrawn the graft easily slips into place. Any wrong position of the graft may be corrected by means of the probe or a small brush. When necessary the flap may be shortened. The whole area of the wound must be covered with strips of skin, which should overlay the edges of the wound, and come together as close as possible, even to the extent of slightly overlapping one another. The skin is now gently pressed in place with a spatula.

The dressing should protect the grafts, and maintain them in position. Better results are obtained by using a moist dressing, changed daily. The vicinity of the wound is smeared with oil to prevent the dressing sticking. The grafts are covered with a strip of protective, soaked in salt solution. Over this is placed a pad of cotton, moistened with salt water; this is covered by a large piece of protective. Over this is another pad, of dry cotton, and the whole is held in place by a cotton bandage, over which a dextrine bandage is applied to prevent slipping. If a dry dressing is to be used, an iodoform dressing is the best. The places from which the skin has been removed are covered with iodoform dust, a dry dressing is applied, and left for a week or two.

Within the first few days the grafts may undergo the following changes: If they are of a pinkish color success is tolerably sure. If they are white they will drop off in a few days. Blood under a graft gives it a bluish color, and endangers the healing process, but does not always lead to suppuration. In order to prevent the entrance of bacteria into the wound, which prevent healing, the dressing should be changed every day during the first week, and the surface irrigated with sterilized salt water.

When the wounded surface is not covered with grafts a fibrinous exudation appears on the free borders, and the grafts begin to separate. The healed grafts become detached, or small epidermal blisters filled with pus appear on the healed spots, and form ulcers that gradually enlarge. The superimposed skin may be broken through from below by granulations, and disappears, temporarily at least, but when the granulations recede later, epidermal islets are again seen. Syphilis may prevent the healing of the grafts.

Plessing gives a series of 40 cases in which transplantation was done 78 times; 17 times on fresh surfaces, and 61 times on scraped granulating surfaces. In 58 cases there was perfect healing, in 12 cases it was incomplete, and in 8 it was a total failure, and the grafting was repeated. The results are better on scraped granulation surfaces than on loose or connective tissue (fascia, periosteum); glandular and muscular tissues give good results. Spongy bone tissue and exposed tendons do not give permanent results. Grafts do not adhere to compact bone.

Plessing lays stress on the following points: Careful disinfection of the hands and instruments, newly prepared sterilized 6 to 1000 salt solution, proper time of operation, thorough hæmostasis, complete covering of the wound with grafts, immobilization of the part, careful bandaging, and daily changes of dressing, with thorough irrigation.

